# Oral cell lysates reduce osteoclastogenesis in murine bone marrow cultures

**DOI:** 10.1007/s10616-024-00688-1

**Published:** 2025-01-08

**Authors:** Layla Panahipour, Azarakhsh Oladzad Abbasabadi, Feng Shao, Reinhard Gruber

**Affiliations:** 1 Department of Oral Biology, University Clinic of Dentistry, Medical University of Vienna, Sensengasse 2a, 1090 Vienna, Austria; 2 Department of Periodontology, School of Dental Medicine, University of Bern, 3010 Bern, Switzerland; 3 Austrian Cluster for Tissue Regeneration, 1200 Vienna, Austria

**Keywords:** Osteoclast, Cell damage, Dental implants, Periodontal treatment, Necrotic cell lysates, Gingival fibroblasts, Epithelial cells, Macrophages, In vitro

## Abstract

Mechanical and thermal cell damage can occur due to invasive procedures related to drilling, the insertion of dental implants, and periodontal treatments. Necrotic cells release the content of their cytoplasm and membrane fragments, thereby signaling the need for repair, which includes bone resorption by osteoclasts and inflammation. Here we screened lysates from human gingival fibroblasts, HSC2 and TR146 oral squamous carcinoma cell lines, as well as murine IDG-SW3 osteocytic and RAW264.7 macrophage cell lines for their potential to modulate in vitro osteoclastogenesis in murine bone marrow cultures. We also tested the impact of necrotic lysates on modulating the expression of inflammatory cues in murine ST2 bone marrow stromal cells. We report here that independent of human or murine origin, all cell lysates significantly reduced in vitro osteoclastogenesis in bone marrow cultures, as indicated by the expression of the osteoclast marker genes cathepsin K and tartrate-resistant acid phosphatase and the respective histochemical staining in multinucleated cells. We also found that lysates from HSC2 and TR146 cells significantly pushed the expression of CCL2, CCL5, CXCL1, IL1, and IL6 in ST2 cells. These findings suggest that oral cell lysates reduce in vitro osteoclastogenesis, but only damaged oral squamous carcinoma cells can force murine stromal cells to produce an inflammatory environment.

## Introduction

Implant dentistry was pioneered by the observations that titanium is an osteoconductive material allowing its stable anchorage in the alveolar bone (Albrektsson 1983). Histological evidence and biomechanical analysis revealed that osseointegration is a biphasic process; this process is initiated by a catabolic phase where the necrotic bone is resorbed and consequently replaced by an anabolic phase where new bone covers the titanium and the bone surface (Vasak et al. 2014). Later on, bone remodeling reinforces and rejuvenates the intimate connection of the dental implant with the local alveolar bone to withstand masticatory forces. In the past, implant research mainly focused on the somewhat predictable anabolic phase of osseointegration, while today, the early catabolic phase receives increasing attention as it affects the transition of primary towards secondary implant stability (Yin et al. 2019; Chen et al. 2019). Histological evidence reveals the presence of osteoclasts resorbing the necrotic bone that occurs upon drilling and implant insertion (Vasak et al. 2014); however, the underlying molecular mechanisms are only beginning to be understood.

Osteoclastogenesis involves the inflammation-related expression of the receptor-activator of the NFkB ligand (RANKL) that links osteocytes (Xiong et al. 2011; Nakashima et al. 2011; Graves et al. 2018) and other cells (Tsukasaki et al. 2018) with bone resorption. In addition, damage-associated molecular patterns (DAMPs) released by serum-starved necrotic osteocytes support osteoclastogenesis (Andreev et al. 2020). Drilling and implant insertion are not restricted to damaged osteocytes; cells residing in the soft tissue are injured and may thus add to the paracrine environment, affecting inflammation and osteoclastogenesis. Moreover, scaling and root planning for the removal of dental plaque and calculus is an invasive procedure that can harm the periodontal soft tissue (Cobb 2002; Trenter and Walmsley 2003), a tissue that is mainly composed of the gingiva is a fibroblast-rich connective tissue covered by the oral epithelium. The overall question arises of how damaged oral cells can incite an inflammatory response within the fibroblast-rich connective tissue that may, in turn, support osteoclastogenesis.

A single-cell transcriptome analysis of oral mucosa in periodontitis patients identifies inflammatory signatures of fibroblastic and epithelial cell populations (Williams et al. 2021). For instance, attracted by chemokine (C–C motif) ligand 2 (CCL2) and other chemokines being highly expressed in gingival fibroblasts (Williams et al. 2021), macrophages infiltrate the connective tissue and contribute to innate immunity, particularly during periodontitis and peri-implantitis (Williams et al. 2021; Carcuac and Berglundh 2014). The same is true for neutrophils attracted by CXCL1 produced by the activated fibroblastic cells (Williams et al. 2021). Fibroblasts also express the pleiotropic pro-inflammatory cytokine IL1 in vitro (Panahipour et al. 2020) and IL6 in periodontitis tissue (Luo et al. 2014). The underlying mechanisms for the increased expression of chemokines and other signaling molecules are not necessarily restricted to septic clues as fibroblasts may also be triggered by damage-associated molecular patterns released upon apoptosis (Lucas et al. 2010), necroptosis (Li et al. 2018), and pyroptosis (Sordi et al. 2023) – or by mechanical and thermal damaged necrotic cells.

Support for this hypothesis comes from our previous research showing that necrotic lysates of HSC2 oral squamous carcinoma cells provoked IL1, IL6, CXCL8, and IL11 expression in gingival fibroblasts– but not vice versa (Sordi et al. 2023; Panahipour et al. 2023). In contrast, however, necrotic lysates from gingival fibroblasts, HSC2, TR146, and RAW264.7 cells reduced the nuclear translocation of p65 in LPS-exposed macrophages (Panahipour et al. 2023). Preliminary data further revealed that lysates prepared from necrotic murine bone marrow stromal ST2 cells are potent inhibitors of osteoclastogenesis (Panahipour et al. 2022). Moreover, exposure of HEK-293 cells to their respective necrotic cell lysates releases clusterin (Rohne et al. 2018), which can inhibit osteoclastogenesis in bone marrow cultures (Choi et al. 2014). Taken together, there is accumulating evidence suggesting that necrotic cells can modulate inflammation and osteoclastogenesis.

This hypothesis prompted us to extend previous observations by testing lysates prepared by sonication and freeze/thawing of human gingival fibroblasts, HSC2 and TR146 oral squamous carcinoma cells, and a murine RAW264.7 macrophage cell line in the respective bioassays (Panahipour et al. 2022). These necrotic cell lysates were tested for their impact on osteoclastogenesis in murine bone marrow cultures (Panahipour et al. 2022). Here, the hematopoietic cells of the bone marrow respond to recombinant RANKL driving the overall sequence of events that culminate in the formation of multinucleated cells staining positive for tartrate-resistant acid phosphatase (TRAP) and express the protease cathepsin K (Suda et al. 1999). We also have implemented murine bone marrow stromal ST2 cells in our analysis as they support osteoclastogenesis in vitro (Udagawa et al. 1989). Our focus is on ST2 cells being capable of expressing chemokines; chemokine (C–C) ligand-2 (CCL2) is important for the formation of osteoclasts (Khan et al. 2016), while CCL5 is more of a suppressor chemokine for osteoclastogenesis (Wintges et al. 2013). Recently, we have shown that TNFα induced the expression of CCL2 and CCL5 in ST2 cells (Panahipour et al. 2022; Kargarpour et al. 2023). Moreover, CCL2 and CCL5 are elevated in gingival crevicular fluid in patients with generalized aggressive periodontitis (Emingil et al. 2004). We now use this bioassay to evaluate the impact of the necrotic lysates on the murine bone marrow culture model of osteoclastogenesis.

Based on these bioassays, we report here that all necrotic cell lysates, with different effects, reduced osteoclastogenesis in bone marrow cultures Still, only necrotic lysates from HSC2 and TR146 oral squamous carcinoma cells could increase the expression of the chemokines in ST2 cells. Thus, necrotic cell lysates can directly reduce the formation of osteoclasts from hematopoietic progenitors and potentially change the paracrine environment of the stromal compartment represented by ST2 cells.

## Methods

### Cell lines

To isolate gingival fibroblasts (GF), tissue explant from the gingiva was cultivated until cells grew and expanded. The tissue was harvested from extracted wisdom teeth considering the approval of the Ethical Committee of the Medical University of Vienna (EK Nr. 631/2007). The oral squamous cell carcinoma cell line (HSC2) was obtained from the Health Science Research Resources Bank (Sennan, Japan), and the buccal squamous cell carcinoma cell line TR146 from the European Collection of Authenticated Cell Cultures (UK Health Security Agency, Salisbury, UK). Macrophage-like cells (RAW264.7) were received from the American Type Culture Collection (LGC Standards GmbH, Wesel, Germany) and the ST2 mesenchymal stromal cell line from Riken Cell Bank (Tsukuba, Japan). All cells were expanded in a growth medium consisting of DMEM, 10% fetal calf serum, and 1% antibiotics (Invitrogen Corporation, Carlsbad, CA, USA). The IDG-SW3 osteocytic cell line (Kerafast, Inc., Boston, MA, USA) was expanded as recommended (31).

### Necrotic cell lysates

Suspensions of GF, HSC2, TR146, RAW264.7, and IDG-SW3 cells at 4 × 10^6^ cells/mL alpha Minimum Essential Medium (αMEM) containing antibiotics were subjected to (i) sonication for three times each time 15 s (Sonoplus; Bandelin electronic GmbH & Co. KG; Berlin, Germany) or (ii) three times freeze-thawing for 8 min at -80 °C and room temperature before centrifugation at 2600 RCF for 5 min (5420; Eppendorf SE, Hamburg, Germany). The supernatants considered as necrotic cell lysate were prepared for each independent experiment.

### Bioassay for osteoclastogenesis

BALB/c mice of 6–8 weeks were received from Animal Research Laboratories (Himberg, Austria). The femora and tibiae were dissected, and the bone marrow was flushed by αMEM supplemented with serum and antibiotics. Bone marrow cells were cultured at 1 × 10^6^ cells/cm^2^ in 24-well plates in a medium supplemented with 20 ng/mL, macrophage-colony stimulating factor (M-CSF; ProSpec, Ness-Ziona, Israel). The following day, the medium was replaced by a growth medium or 100% cell lysates, each supplemented with 30 ng/mL RANKL, 20 ng/mL M-CSF, and 10 ng/mL transforming growth factor-β1 (all ProSpec). Medium change was performed on day 3 and day 5. On day 8, histochemical staining for tartrate-resistant acid phosphatase (TRAP) was done (Sigma Aldrich, St. Louis, MO). Microscopical images were taken by a light microscope (Oxion fluorescence, Euromex, Arnheim, Netherlands). RT-PCR detected the transcription levels of TRAP and cathepsin K (CTSK). Total RNA was extracted (ExtractMe total RNA kit, Blirt S.A., Gdańsk, Poland) and complementary DNA was produced (LabQ, Labconsulting, Vienna, Austria). Amplification was done one on a CFX Connect™ Real-Time PCR Detection System (Bio-Rad Laboratories, Hercules, CA). Primers sequences were CTSK-F: TGTATAACGCCACGGCAAA, CTSK-R: GGTTCACATTATCACGGTCACA; TRAP-F: AAGCGCAAACGGTAGTAAGG, TRAP-R: CGTCTCTGCACAGATTGCAT, GAPDH‐F: AACTTTGGCATTGTGGAAGG, GAPDH‐R: GGATGCAGGGATGATGTTCT. Transcription levels were calculated by the ΔΔCt method. Data are expressed as an x-fold change normalized to the unstimulated cells.

### Bioassay for chemokine and cytokines expression

ST2 were seeded at 3 × 10^5^ cells/cm^2^ and, on the next day, exposed to αMEM containing serum and antibiotics or the respective necrotic cell lysates. After 16 h, total RNA was extracted, complementary DNA was synthesized, and polymerase chain reaction was performed using the following primers: CCL2-F: GCTACAAGAGGATCACCAGCAG, CCL2-R: GTCTGGACCCATTCCTTCTTGG, CCL5-F: CCTGCTGCTTTGCCTACCTC, CCL5-R: ACACACTTGGCGGTTCCTTC, CXCL1-F: AGACTCCAGCCACACTCCAA, CXCL1-R: TGACAGCGCAGCTCATTG, IL1-F: TTGGTTAAATGACCTGCAACA, IL1-R: GAGCGCTCACGAACAGTTG, IL6-F: GCTACCAAACTGGATATAATCAGGA, IL6-R: CCAGGTAGCTATGG-TACTCCAGAA. All expressions were normalized to GAPDH by the ΔΔCt method. Data are expressed as an x-fold change over the unstimulated control.

### Statistical analysis

The experiments were repeated at least three times, and every data point belonged to an independent experiment. Statistical analysis was performed using Prism v9 (GraphPad Software, La Jolla, CA, USA).

## Results

**Fig. 1:**
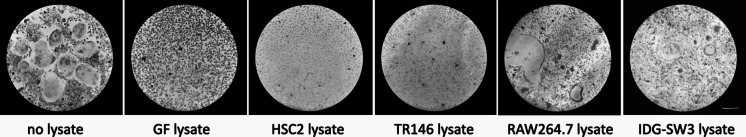
Sonicated cell lysates reduce the formation of TRAP-positive cells in murine bone marrow cultures. Primary bone marrow cells were grown in the presence of RANKL, MCSF, and TGF-β1 with and without necrotic cell lysates prepared by sonication. Histochemical staining represents the activity of TRAP, which is mainly produced by multinucleated cells. Note that cell lysates diminished the number and lowered the size of the remaining TRAP-positive cells. The scale bar indicates 100 µm

First insights revealed that necrotic lysates prepared from the ST2 cell line can inhibit in vitro osteoclastogenesis (Panahipour et al. 2022). To determine whether necrotic lysates from other cells representing the oral soft tissue and osteocytes modulate osteoclastogenesis, murine bone marrow cells were exposed to the respective lysates. These experiments revealed that osteoclastogenesis was successfully initiated by RANKL, M-CSF, and TGF-β1 as shown by the formation of multinucleated cells staining positive for TRAP (Fig. 1). Upon sonication, necrotic lysates from gingival fibroblasts, HSC2, TR146, RAW264.7 and IDG-SW3 cells, all caused an apparent reduction of osteoclastogenesis indicated by the almost lack of TRAP-positive large multinucleated cells (Fig. 1). The lysates prepared from RAW264.7 macrophages caused less multinucleated TRAP-positive cells but allowed osteoclastogenesis to occur (Fig. 1).

**Fig. 2:**
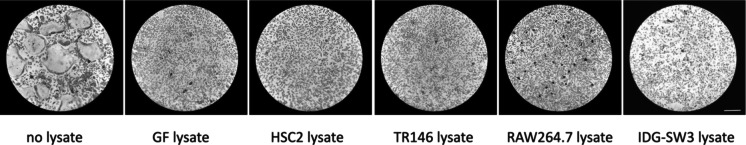
Freeze/thawing cell lysates reduce the formation of TRAP-positive cells in murine bone marrow cultures. Primary bone marrow cells were grown with RANKL, MCSF, and TGF-β1 in the presence and absence of cell lysates prepared by three cycles of freeze/thawing. Histochemical staining represents the activity of TRAP, which is mainly produced by multinucleated cells. Necrotic cell lysates reduced the number and size of the remaining TRAP-positive cells. The scale bar shows 100 µm

Next, we exposed the cell suspension to three repeated cycles of freeze/thawing. We observed that consistent with lysates prepared by sonication, the necrotic lysates prepared by freeze/thawing of gingival fibroblasts, HSC2, TR146, and IDG-SW3 were strong in their potential to reduce in vitro osteoclastogenesis (Fig. 2). RAW264.7 had the least potential to reduce osteoclastogenesis, showed by the presence of small multinucleated TRAP-positive cells (Fig. 2).

**Fig. 3:**
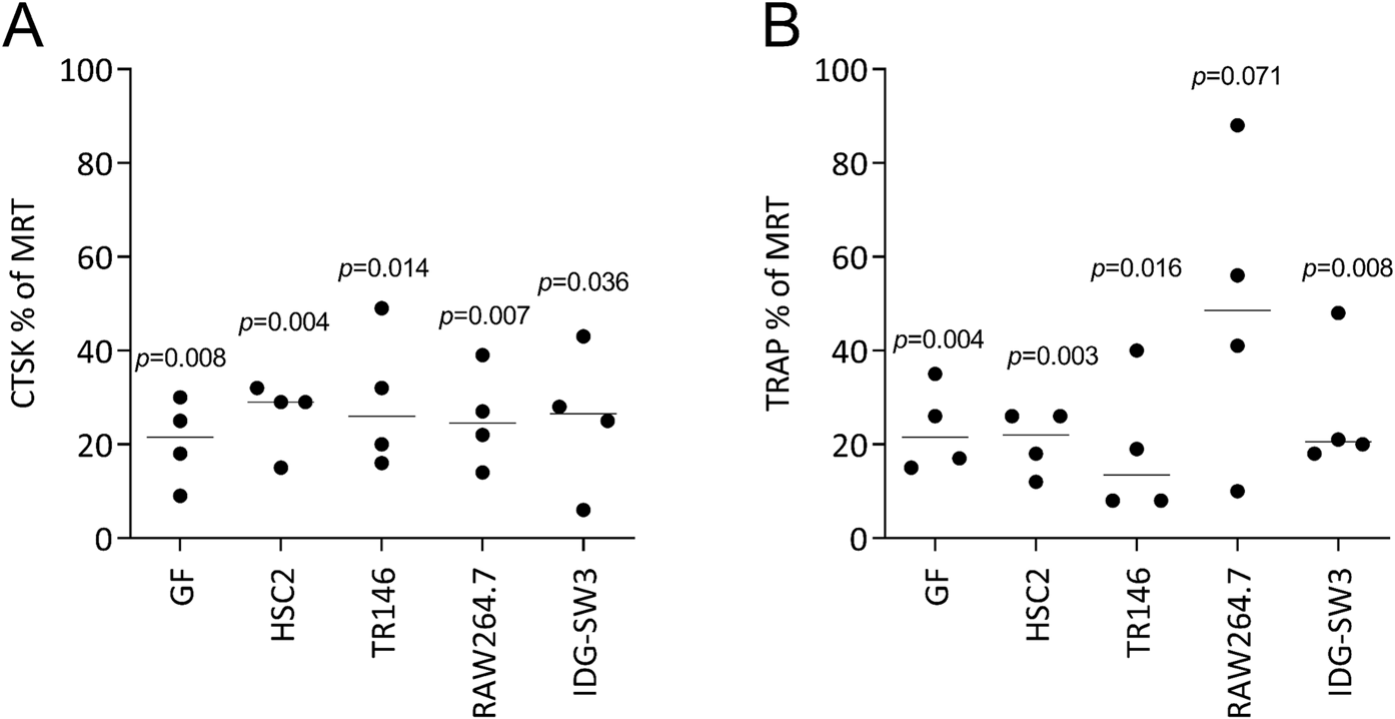
Sonicated cell lysates reduce the expression of osteoclast marker genes. Primary bone marrow cells were cultured in the presence of RANKL, MCSF, and TGF-β1 (MRT) with and without cell lysates prepared by sonication. Data show the percent (%) changes in (A) cathepsin K (CTSK) expression and (B) tartrate-resistant acid phosphatase (TRAP) compared to unstimulated cells. Data points indicate independent experiments. Statistical analysis was based on a ratio-paired t-test

**Fig. 4:**
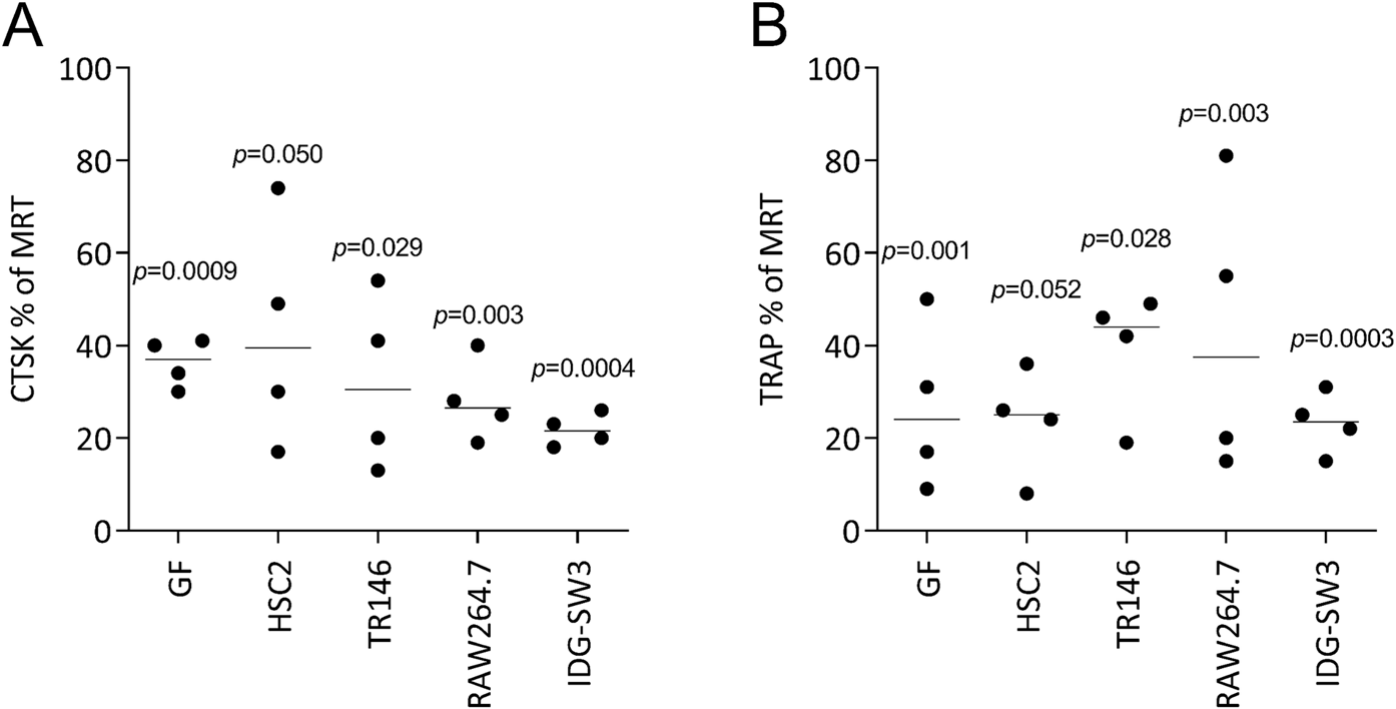
Freeze/thawing lysates reduce the expression of osteoclast marker genes. Primary bone marrow cells were cultured in the presence of RANKL, MCSF, and TGF-β1 (MRT) with and without cell lysates prepared by sonication. Data show the percent (%) changes in (A) cathepsin K (CTSK) expression and (B) tartrate-resistant acid phosphatase (TRAP) compared to unstimulated cells. Data points indicate independent experiments. Statistical analysis was based on a ratio-paired t-test

We further addressed the impact of the various cell lysates on the expression of osteoclast marker genes similar to what we have reported for ST2 cell lysates (Panahipour et al. 2022). Gene expression analyses showed that the presence of necrotic lysates prepared by sonication of gingival fibroblasts, HSC2, TR146, and IDG-SW3 cells, all caused a reduced expression of CTSK and TRAP. The sonicated lysates prepared from RAW264.7 macrophages failed to reach the level of significance when testing for TRAP (Fig. 3). Consistently, the lysates prepared by freeze/thawing of gingival fibroblasts, HSC2, TR146, and IDG-SW3 cells reduced osteoclastogenesis; with lysates from RAW264.7 macrophages, we noticed a stronger variation in TRAP marker genes compared to the necrotic lysates prepared by the other cells (Fig. 4).

Primary bone marrow cells were grown in the presence of RANKL, MCSF, and TGF-β1 (MRT) with and without cell lysates prepared by freeze/thawing. Data show the percent (%) changes in (A) cathepsin K (CTSK) expression and (B) tartrate-resistant acid phosphatase (TRAP) compared to unstimulated cells. Data points indicate independent experiments. Statistical analysis was based on a ratio-paired t-test.

**Fig. 5:**
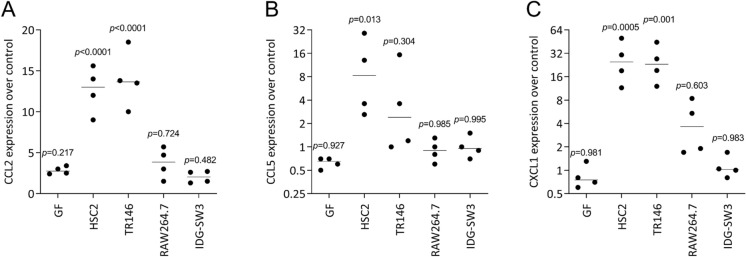
Sonicated HSC2 and TR146 cell lysates increase CCL2, CCL5 and CXCL1 expression in ST2 cells. ST2 cells were cultured in the presence of cell lysates prepared by sonication. Data show the percent x-fold changes in the expression of (A) CCL2, (B) CCL5, and (C) CXCL1 compared to unstimulated cells. Data points indicate independent experiments. Statistical analysis was based on an unpaired ordinary one-way ANOVA

**Fig. 6:**
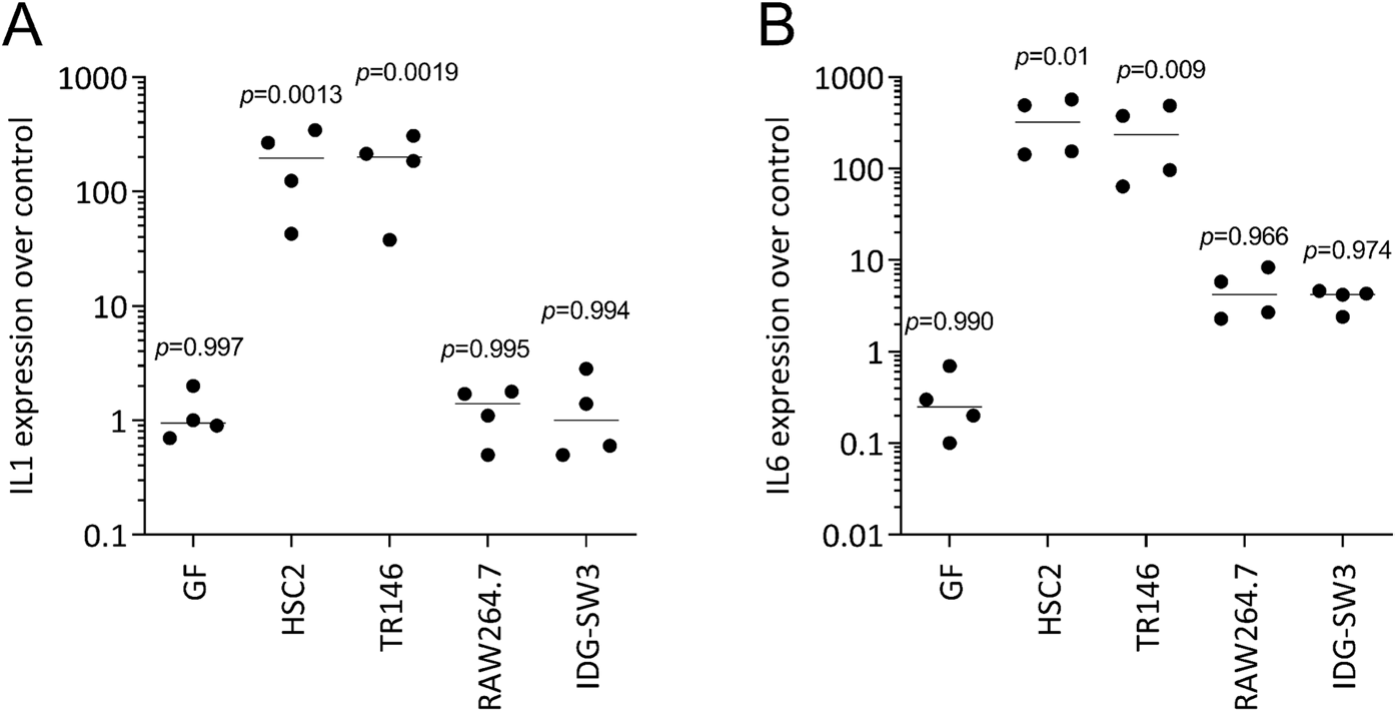
Sonicated HSC2 and TR146 cell lysates increase IL1, and IL6 expression in ST2 cells. ST2 cells were cultured in the presence of cell lysates prepared by sonication. Data show the percent x-fold changes in the expression of (A) CCL2, (B) CCL5, and (C) CXCL1 compared to unstimulated cells. Data points indicate independent experiments. Statistical analysis was based on an unpaired ordinary one-way ANOVA

Finally, we simulated the paracrine environment capable of modulating osteoclastogenesis by implementing ST2 bone marrow stromal cells (Udagawa et al. 1989). We asked if the necrotic lysates can change the expression of chemokines affecting osteoclastogenesis. We show here that it is mainly the cell lysates from HSC2 cells and TR146 that increased the basal expression of CCL2, the agonist of osteoclastogenesis (Khan et al. 2016), but also, though less pronounced, CCL5, a suppressor chemokine for osteoclastogenesis (Wintges et al. 2013) as well CXCL1 (Poli et al. 1994; Hardaway et al. 2015) (Fig. 5). Additionally, we observed a robust increase in the expression of IL1 and IL6 in ST2 cells when exposed to lysates from HSC2 cells and TR146, and IL1 and IL6 are modulators of osteoclastogenesis (Poli et al. 1994; Hardaway et al. 2015) (Fig. 6). Thus, these data suggest that necrotic human epithelial cell lines can target the murine stromal cells to change the expression of chemokines and cytokines, potentially affecting osteoclastogenesis.

ST2 cells were cultured in the presence of cell lysates prepared by sonication. Data show the percent x-fold changes in expression of (A) IL1 and (B) IL6 compared to unstimulated cells. Data points indicate independent experiments. Statistical analysis was based on an unpaired ordinary one-way ANOVA.

## Discussion

This study was motivated by how damaged necrotic cells affect the paracrine environment using osteoclastogenesis and chemokine expression as a bioassay. Bone resorption occurs early upon implant insertion to remove the necrotic bone, mainly the dying osteocytes driving this catabolic event (Vasak et al. 2014; Chen et al. 2019). Bone resorption linked to periodontitis and peri-implantitis is a consequence of chronic inflammation (Taubman et al. 2005); but may also be affected by clinical interventions that harm the periodontal cell – the gingival fibroblasts, the oral epithelial cells, and the macrophages accumulating in the inflamed periodontal soft tissue (Williams et al. 2021). Considering this clinical scenario, we have recently reported that lysates prepared from the murine bone marrow stromal cell line ST2 attenuate in vitro osteoclastogenesis (Panahipour et al. 2022). We extend this research by implementing cells representing the oral soft tissue and an osteocytic cell line. The significant finding of this research was that (i) lysates prepared from gingival fibroblasts, oral epithelial cells, and IDG-SW3 cells consistently reduced osteoclastogenesis in vitro. Lysates prepared by RAW264.7 macrophages are less potent and restrain the overall conclusion that damaged necrotic oral cells possess a paracrine activity capable of blocking osteoclastogenesis in vitro. Another observation was that (ii) exclusively lysates from HSC2 cells and TR146 were capable of provoking a robust increase of inflammatory cues being expressed in murine ST2 cells. Thus, there are two possible modes of action – the direct effect by blocking osteoclastogenesis in bone marrow cultures and the indirect one by changing the paracrine environment of stromal cells.

If we relate our first main observation to other studies, and apart from our previous research (Panahipour et al. 2022), we find support for our observations coming from lysates prepared from HEK-293 cells releasing clusterin (Rohne et al. 2018), and clusterin can reduce osteoclastogenesis in vitro (Choi et al. 2014). In contrast to our findings, however, supernatants prepared from serum-starved necrotic IDG-SW3 cells increased osteoclastogenesis via a mincle-dependent mechanism (Andreev et al. 2020). There is thus a discrepancy between supernatants harvested from serum-starved necrotic cells and the total necrotic cell lysates, the first stimulating while the latter inhibiting in vitro osteoclastogenesis, respectively. Surprisingly, and even though the environmental changes are relevant in osteoclastogenesis, for instance, the bidirectional ephrinB2-EphB4 signaling (Zhao et al. 2006), studies on how cell lysates regulate osteoclastogenesis are rare. We can only speculate about the molecular mechanism for decreasing osteoclastogenesis in murine bone marrow cultures. One possible pathway is that cell lysates contain IFN-β produced by osteocytes, and IFN-β is known to inhibit osteoclastogenesis (Hayashida et al. 2014). However, most of the inhibitor cytokines are produced by lymphocytes, macrophages, and dendritic cells (Amarasekara et al. 2018). Alternatively, our necrotic lysates may contain proteases inactivating the osteoclast-induction cocktail of RANKL, M-CSF, and TGF-β1; however, usually, proteases release membrane-bound RANKL (Lum et al. 1999) and activate TGF-β1 (Jenkins 2008). Another option would be the lysates containing molecules adsorbing, thereby neutralizing the osteoclast-induction cocktail; for instance, collagen can adsorb TGF-β1 (Stahli et al. 2016), but overall, the mechanism of how cell lysates reduce osteoclastogenesis in murine bone marrow cultures remains unclear.

Damage-associated molecular patterns (DAMPs) provide another interesting line of research. DAMPs are molecules released from damaged or dying cells causing an innate immune response (Hudson and Lippman 2018). DAMPs include but are not limited to extracellular matrix components (Frevert et al. 2018) as S100 and heat shock proteins – all of which bind and active TLRs and other pattern recognition receptors (Gong et al. 2020) – and, importantly, lipopolysaccharides (LPS) from E. coli (*Escherichia coli*) as a TLR4 agonist significantly inhibits osteoclastogenesis in murine in vitro cultures (Takami et al. 2002; Muller et al. 2017). Thus, there is a rationale for DAMPs activating TLR4 signaling in bone marrow cells being responsible for blocking osteoclastogenesis. However, this is unlikely as the respective lysates reduce the LPS-induced inflammatory response of RAW264.7 macrophages in vitro (Panahipour et al. 2023). Thus, the molecular mechanisms of how the lysates reduce in vitro osteoclastogenesis remain puzzling.

Our second main observation was that lysates from HSC2 and TR146 greatly induced the expression of CCL2, CCL5, CXCL1, IL1, and IL6 in murine ST2 cells – neither lysates from gingival fibroblasts nor those from IDG-SW3 osteocytic cells were capable of provoking such an increase. Most of the inflammatory cues, such as CCL2 (Khan et al. 2016), CXCL1 (Hardaway et al. 2015), IL1 (Polzer et al. 2010), and IL6 (Poli et al. 1994), are potent inducers of osteoclastogenesis; only CCL5 is more of a suppressor for osteoclastogenesis (Wintges et al. 2013). These findings are consistent with our observations showing that lysates of HSC2 and TR146 cells are stimulating the expression of cytokines and CXCL8 in gingival fibroblasts (Sordi et al. 2023). The question of the underlying molecular mechanism remains unclear and might involve TLR activation; for instance, the TLR2 agonists Pam3CSK4 can increase cytokine expression in ST2 cells (Kargarpour et al. 2023). Considering that TLR2 is sensitive to extracellular matrix components, histones, high mobility group box 1 (HMGB1), and other DAMPs (Frevert et al. 2018), it can be speculated that DAMPs released by HSC2 and TR146 may have caused the increase in chemokine and cytokine expression. The question remains: Why don’t DAMPs from gingival fibroblasts or those from IDG-SW3 have this activity? Thus, our preliminary data might help establish a fibroblast-based bioassay to screen for DAMP being released from necrotic oral epithelial cells.

The observation that ST2 cells increasingly expressed CCL2 (Khan et al. 2016), CXCL1 (Hardaway et al. 2015), IL1 (Zwerina et al. 2007), and IL6 (Poli et al. 1994), all agonists of osteoclastogenesis and to some extent also CCL5, an antagonist of osteoclastogenesis (Wintges et al. 2013) is maybe less surprising since CCL2, CCL5, and IL6 are expressed in response to TNFα in ST2 cells (Panahipour et al. 2022; Kargarpour et al. 2023). The bioassay, therefore, has to be interpreted not strictly related to osteoclastogenesis but more in the direction that lysates can change the paracrine environment of stromal cells, an environment that affects the mobilization and differentiation of hematopoietic cells, including neutrophils and macrophages, as well as lymphocytes. For instance, CCL2 and CCL5 are elevated in gingival crevicular fluid in patients with generalized aggressive periodontitis (Emingil et al. 2004), and CCL2 is increasingly expressed in fibroblasts from periodontitis patients (Williams et al. 2021). Likewise, CCL2 and CCL5 are elevated in other diseases, such as pulmonary sarcoidosis (Palchevskiy et al. 2011) or the synovial fluid of patients with juvenile rheumatoid arthritis (Yao et al. 2006). Thus, our findings that HSC2 and TR146 lysates increase CCL2 and CCL5 in ST2 might be linked to the recruitment and activation of monocytes and macrophages. In support of this hypothesis, the enhanced CXCL1 expression points towards the recruitment of neutrophils (Kobayashi 2008). IL1 and IL6 have pleiotropic pro-inflammatory functions (Mbalaviele et al. 2017; Tanaka et al. 2014), similarly changing the paracrine environment of stromal cells.

This discussion clearly shows the study’s limitation, namely that the findings on osteoclastogenesis but also those related to the chemokine and cytokine expression in ST2 cells are descriptive, and we are left with many questions about how to explain these observations on a molecular and clinical level. One question is, if the in vitro observation has relevance in vivo – and as we are interested in dentistry, the clinical impact of the findings in the oral cavity. We are left with another open question of how invasive dental treatments affect cell necrosis and, if this translates into reducing osteoclastogenesis in vivo. Moreover, we can ask if the necrotic epithelial cells – here oral squamous carcinoma cells—change the local stromal environment towards a CCL2 and CCL5-mediated migration of monocytes, and CXCL1-mediated migration of neutrophils. Our future research also relates to a possible hierarchy of the expressed genes; for instance, the increasingly expressed inflammatory cytokines IL1 and IL6 might drive the CCL2, CCL5, and CXCL1 expression. It can thus not be ruled out that it is IL1, IL6, and possibly other mediators being increased in ST2 cells, that in turn, drive the chemokine expression. Thus, HSC2 and TR146 lysates might indirectly affect the autocrine environment of ST2 cells, causing changes in chemokine expression. Regardless of whether chemokine expression is a direct result of cytokine activity or both are independently induced by HSC2 and TR146 lysates, our preliminary findings support the fundamental concept that fibroblasts, such as ST2 cells, can transform into inflammatory cells in a catabolic environment. These cells express chemokines and cytokines thereby attracting and activating neutrophils, lymphocytes, and antigen-presenting cells (Williams et al. 2021), a process that is presumably happening during transient inflammation, before its resolution, in the context of tissue regeneration (Schett and Neurath 2018).

However, care should be taken when interpreting findings as the necrotic cell lysate is a sonicated living cell. Thus, the process of necrosis in these cells is not clearly present. Moreover, the use of HSC2 and TR146 cancer cells does not necessarily reflect the normal oral epithelial cells; thus, future research should test the activity of the respective lysates in the bioassays we have performed. It would be interesting to understand what cellular components the HSC2 and TR146 cells release, in contrast to the gingival fibroblasts and the other cell lines, that provoke the inflammatory response in the ST2 mouse stromal cells – while most lysates significantly reduce in vitro osteoclastogenesis in mouse bone marrow cultures. Considering these limitations and the pilot nature of the research, our study incites the curiosity to uncover the mechanisms of how necrotic cell lysates affect osteoclastogenesis and the local response of the fibroblastic stromal environment. This research line is of potential clinical relevance considering the invasive procedures in dental treatments and possibly in other fields of regenerative medicine.

## Data Availability

No datasets were generated or analysed during the current study.
